# Maternal educational level and children’s healthy eating behaviour: role of the home food environment (cross-sectional results from the INPACT study)

**DOI:** 10.1186/s12966-014-0113-0

**Published:** 2014-09-12

**Authors:** Wilke JC van Ansem, Carola TM Schrijvers, Gerda Rodenburg, Dike van de Mheen

**Affiliations:** IVO Addiction Research Institute, Heemraadssingel 194, Rotterdam, DM 3021 The Netherlands; Erasmus Medical Centre, Postbox 2040, Rotterdam, CA 3000 The Netherlands; Department of Health Education and Promotion, Maastricht University, Maastricht, The Netherlands

**Keywords:** Fruit and vegetable consumption, Breakfast consumption, Socio-economic status, Home food environment, Children, Parents

## Abstract

**Background:**

The aims of this study are 1) to investigate the association between maternal educational level and healthy eating behaviour of 11-year-old children (fruit, vegetables and breakfast consumption), and 2) to examine whether factors in the home food environment (parental intake of fruit, vegetables and breakfast; rules about fruit and vegetables and home availability of fruit and vegetables) mediate these associations.

**Methods:**

Data were obtained from the Dutch INPACT study. In total, 1318 parent–child dyads were included in this study. Multilevel regression models were used to investigate whether factors of the home food environment mediated the association between maternal educational level and children’s healthy eating behaviour.

**Results:**

Children of mothers with a high educational level consumed more pieces of fruit per day (B = 0.13, 95% CI: 0.04-0.22), more grams of vegetables per day (B = 23.81, 95% CI = 14.93-32.69) and were more likely to have breakfast on a daily basis (OR = 2.97, 95% CI: 1.38-6.39) than children of mothers with a low educational level. Home availability, food consumption rules and parental consumption mediated the association between maternal education level and children’s fruit and vegetable consumption. Parental breakfast consumption mediated the association between maternal education level and children’s breakfast consumption.

**Conclusions:**

Factors in the home food environment play an important role in the explanation of socio-economic disparities in children’s healthy eating behaviour and may be promising targets for interventions.

## Background

Dietary behaviour is important for the development and growth of children and also influences health outcomes later in life. Fruit and vegetables and daily breakfast consumption are important components of a healthy diet and their beneficial effects on health are well documented. Diets rich in fruit and vegetables protect against cardiovascular disease (CVD), some types of cancer, and obesity [1,2]. Regular breakfast consumption is associated with better cognitive performance and a reduced risk of becoming overweight or obese among children and adolescents [3,4]. Despite the importance of healthy dietary behaviours, the majority of the children in the Netherlands, as in other countries, does not consume the recommended amounts of fruit and vegetables [5-8]. In addition, breakfast skipping is highly prevalent in Europe and the United States [9,10]. Also, because dietary habits track into adulthood, it is important to develop interventions aimed to improve dietary behaviours of children [11,12].

Children and adolescents with a low socio-economic status (SES) consume less fruit and vegetables than children and adolescents with a high SES [13-16]. Furthermore, a Norwegian study found an increase in socio-economic disparities in adolescent’s fruit and vegetable consumption between 2001 and 2008 [17]. Studies of socio-economic disparities in breakfast consumption showed inconsistent findings. A literature review found that parental educational level and parental unemployment were unrelated to adolescents and children’s breakfast consumption [18]. However, other studies found a positive association between maternal educational level and children’s breakfast consumption [9,19,20]. Given the inconsistencies in the findings from previous studies and the relative small part of the literature assessing socio-economic disparities in dietary behaviour of children, the first aim of this study is to investigate socio-economic differences in healthy eating behaviours of children (fruit, vegetable and breakfast consumption).

The home food environment is important in the development of children’s dietary behaviour [21]. Parents have an important influence on the dietary behaviour of children because they generally determine which food is available at home, they can set rules about what their children are allowed to eat and they act as role models, also with respect to dietary behaviour [22]. Several literature reviews concluded that aspects of the home environment are associated with children’s fruit and vegetable intake [16,23,24]. Home environmental factors found to be positively related to children’s fruit and vegetable intake are home availability, family rules and parental intake. For breakfast consumption, parental breakfast consumption is an important home environmental factor that is positively associated with children’s breakfast consumption [18].

**Figure 1:**
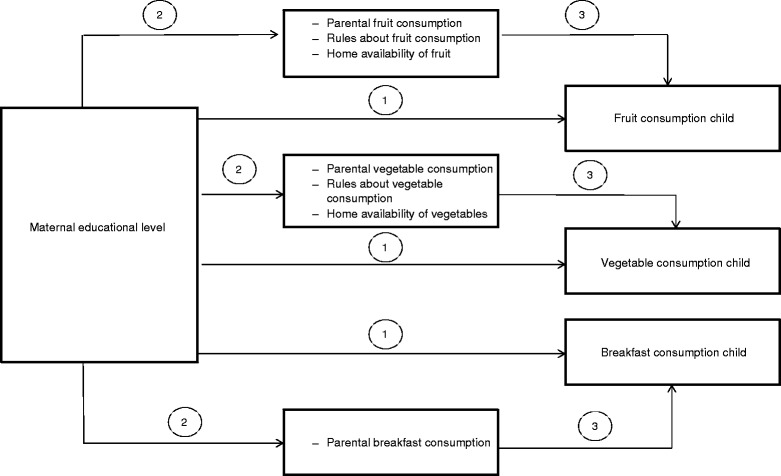
**Research model.**

As stated before, the first aim of this study is to investigate socio-economic differences (maternal educational level is used as indicator for children’s SES) in healthy eating behaviours of children. However, SES does not directly influence dietary behaviour and is not a modifiable correlate of children’s dietary behaviour. Thus it is important to identify modifiable determinants that may explain the socio-economic disparities in children’s healthy eating behaviour. Therefore, the second aim of this study is to examine whether factors in the home food environment (parental intake of fruit, vegetables and breakfast; rules about fruit and vegetables and home availability of fruit and vegetables) mediate the association between maternal educational level and children’s healthy eating behaviours (fruit, vegetable and breakfast consumption). Figure 1 presents the research model.

## Methods

### Study population and design

The data used in this study are derived from the Dutch INPACT study, INPACT being the acronym for IVO Nutrition Physical Activity Child cohort. This longitudinal study among 8 to 12 year olds and their parents investigated modifiable environmental determinants of children’s dietary behaviour. Participants of the INPACT study were recruited through primary schools in the southern part of the Netherlands (Eindhoven and surroundings). The municipal health service invited all general primary schools (n = 265) in this area to participate in this study. Ninety one schools (34.3%) agreed. The response rate of schools in rural and urban areas was similar. A sample of 1844 parent–child dyads (62.5%) gave informed consent. Trained research assistants visited the participating primary schools and measured children’s height and weight. Children completed a short questionnaire at school and parents completed a questionnaire at home. The questionnaire topics varied annually. The INPACT study was approved by the Medical Ethical Committee at Erasmus Medical Centre, Rotterdam. The present study was based on cross-sectional data collected in the last wave (2011), in which a questionnaire was completed by 1428 primary caregivers. In most cases (n = 1312, 92.1%) the primary caregiver was the mother.

### Measurements

#### Socio-economic status

The three most commonly used indicators of SES are educational level, income and occupation [25]. Of these three SES indicators, educational level was found to be the strongest and most consistent in predicting health behaviour [25]. In this study, maternal educational level was used as an indicator of children’s SES because several studies found maternal educational level to be a reliable determinant of children’s dietary behaviour and childhood obesity [9,19,20]. In addition, traditionally, in the majority of the households the mother provides the food for the family and, therefore, maternal educational level also impacts the dietary behaviour of the other members of the family and the home availability of foods. Maternal educational level was classified into three groups: ‘low educational level’ (primary school and lower secondary education), ‘intermediate educational level’ (intermediate vocational level, higher secondary school and pre-university education) and ‘high educational level’ (higher vocational education and university). Throughout the remainder of this paper we thus refer to ‘low SES’ (children of mothers with a low educational level), ‘intermediate SES’ (children of mothers with an intermediate educational level) and ‘high SES’ (children of mothers with a high educational level).

#### Outcome measures (children’s fruit, vegetable and breakfast consumption)

Children’s fruit, vegetable and breakfast consumption were measured with a questionnaire based on a validated Food Frequency Questionnaire [26-29]. Parents reported how many days in a normal week their child consumed 1) fruit (fresh or canned fruit), 2) cooked, fried, steamed or otherwise heated vegetables, 3) salad or other raw vegetables and 4) breakfast. Answering categories ranged from ‘none or less than one day a week’ to ‘7 days a week’.

Additionally, parents reported the numbers of servings of fruit and vegetables consumed by their child on such a day. For fruit, answer categories ranged from ‘0 pieces a day’ to ‘more than 3 pieces a day’, by increments of half a piece of fruit. Reported fruit consumption of more than 3 pieces a day (n = 4) was recoded as ‘4 pieces a day’. For vegetables, answer categories ranged from ‘0 serving spoons’ to ‘more than 4 serving spoons a day’, by increments of half a serving spoon. Reported vegetable consumption of more than 4 serving spoons (n = 12) was recoded as ‘5 serving spoons a day’. One serving spoon of vegetables was equivalent to 50 grams of vegetables. Total vegetable consumption was calculated in grams for each child by multiplying consumption frequency (how many days a child consumed vegetables) and serving spoons of vegetables. Subsequently, the vegetable consumption was converted to an amount consumed in a day. Total fruit consumption was calculated in pieces for each child by multiplying consumption frequency (how many days a child consumed fruit) and servings (pieces of fruit). Children’s fruit consumption was also converted to an amount consumed in a day.

Breakfast consumption was dichotomized into ‘daily’ and ‘not daily’, due to limited variation in the answering categories.

#### Potential mediating variables

##### Parental intake of fruit, vegetables and breakfast

Parental fruit, vegetable and breakfast consumption were measured and calculated in the same way as children’s fruit, vegetable and breakfast consumption.

##### Rules about fruit and vegetable consumption

We assessed whether parents set rules regarding their child’s fruit and vegetable consumption with the following questions: ‘Do you have the rule that your child should eat 2 pieces of fruit a day?’ and ‘Do you have the rule that your child should eat 200 grams of vegetables a day?’ These specific amounts of fruit and vegetables are consistent with the Dutch guidelines for fruit and vegetables [30]. Response categories were ‘yes’ and ‘no’. These questions were derived from the ENDORSE study [31].

##### Home availability of fruit and vegetables

The availability of fruit and vegetables at home was measured using a questionnaire based on the validated Home Environment Survey [32]. Parents were asked about the availability of 1) fruit and 2) vegetables in their home. Response categories were ‘yes, always’, ‘yes, usually’, ‘sometimes’, ‘no, usually not’ and ‘no, never’. Due to limited variability of these variables, we dichotomized both variables into ‘always’ (‘yes, always’) and ‘not always’ (‘yes, usually’; ‘sometimes’; ‘no, usually not’; ‘no, never’).

#### Potential confounders

The following variables are considered as potential confounders: age, gender, ethnicity and body mass index (BMI) of the child. Age, gender and ethnicity of the child were reported by the parents. A child’s age (in years) was calculated on the basis of the date of birth and the date of measurement. For the purpose of analysis we dichotomised child’s age into ‘≤ 11 years’ versus ‘> 11 years. Children’s ethnicity was categorised into ‘Dutch native’ (both parents born in the Netherlands) and ‘immigrants’ (at least one of the parents was born outside the Netherlands). Children’s body mass index (BMI) was calculated on the basis of weight and height, which were measured with clothes but without shoes to the nearest 0.1 kg and 0.1 cm; the measurements were made by trained research assistants. BMI cut-off points for children were used to define overweight and obesity [33]. Subsequently child BMI was dichotomised into ‘overweight’ (‘overweight’ and ‘obesity’) versus no overweight (‘underweight and normal weight’).

### Data analysis

Respondents who lacked data for maternal educational level were excluded from this study (n = 110, 7.7%). In total, 1318 children and parents were included in this study. Descriptive analyses were performed to describe the characteristics of the study population.

To investigate whether home environmental factors mediated the association between maternal educational level and children’s healthy eating behaviour, we used Baron and Kenny’s four-step approach [34]. According to Baron and Kenny, there are three criteria for mediation: 1) the predictive variable has to be associated with the outcome variable, 2) the predictive variable has to be associated with the mediator, and 3) the mediator has to be associated with the outcome variable (adjusted for the predictive variable). If all the associations assessed in steps 1–3 are statistically significant, the criteria for mediation have been met. Step four of the approach is to test the mediation model: mediation is supported if the association between the predictive variable and the outcome variable changes after controlling for the mediator.

For each outcome measure (child fruit intake, child vegetable intake and child breakfast consumption) the steps of the mediation approach were conducted separately. Depending on the scale of the outcome measures, logistic regression models or linear regression models were used to test the subsequent steps of the mediation- approach.

Several potential mediators were tested for the outcome measures ‘child’s fruit consumption’ and ‘child’s vegetable consumption’. If it appeared that more than one potential mediator met the criteria for mediation, the unique contribution of each mediator was determined (single mediator model). Next, a multivariate mediation model was tested. Bootstrapping resampling techniques were used to calculate confidence intervals for the mediated effects.

All regression models were adjusted for the potential confounders. Due to the used sample-strategy (children were recruited trough schools), the data have a nested structure (children within schools). To take into account potential clustering effects, we investigated the associations using multilevel regression analyses. Analyses were performed using R (2013). Cases with missing values were removed per analysis. Due to missing values the computed models for fruit, vegetable and breakfast consumption are based on different numbers of participants.

## Results

Background characteristics of the study population are presented in Table 1. Mean age of the children was 11 years, the majority was native Dutch and not overweight, and about half were boys. Significant differences between the three educational levels were found in the background characteristics: relative to the high and low SES groups there were more girls in the intermediate SES group. More children in the low SES group were overweight, were older than 11 years, and were immigrants compared with children in the intermediate and high SES groups.

**Table 1:** **Characteristics of the study population: total sample and sample according to socio-economic status (SES)**

	***Total sample***	***Low SES***	***Intermediate SES***	***High SES***	***P-value***
**Mean age N (%)**	N = 1317	N = 263	N = 628	N = 426	**0.00**
≤11 years	1119 (85.0)	205 (77.9)	528 (84.1)	386 (90.6)	
> 11 years	198 (15.0)	58 (22.1)	100 (15.9)	40 (9.4)	
**Gender %**	N = 1318	N = 263	N = 629	N = 426	**0.02**
Boys	50.8	52.5	46.7	55.6	
Girls	49.2	47.5	53.3	44.4	
**Child’s BMI %**	N = 1283	N = 252	N = 616	N = 415	**0.01**
Overweight	11.2	16.7	10.6	8.9	
No overweight	88.8	83.3	89.4	91.9	
**Child’s ethnicity %**	N = 1318	N = 263	N = 629	N = 426	**0.02**
Native Dutch	88.8	84.4	90.9	89.4	
Immigrant	11.2	15.6	9.1	10.6	

### Fruit consumption

Table 2 provides data on children’s fruit consumption and determinants of children’s fruit consumption stratified by SES. Children with a low SES had the lowest fruit consumption (on average 0.96 pieces per day) while children with a high SES had the highest fruit consumption (on average 1.07 per day). Table 3 presents data on the association between children’s SES and their fruit consumption. Children with a high SES consumed more fruit than children with a low SES (B = 0.13, 95% CI: 0.04-0.22). There was no significant difference in fruit consumption between children with an intermediate SES and those with a low SES.

**Table 2:** **Descriptives of the key study variables**

	**Socio-economic status (SES)**		
	**Low**	**Intermediate**	**High**
***Fruit (N = 1269)***	N = 247	N = 607	N = 415
*Children’s fruit intake, pieces per day (mean, SD)*	0.96 (0.65)	0.99 (0.57)	1.07 (0.60)
*Parental fruit intake, pieces per day (mean, SD)*	0.97 (0.74)	1.04 (0.74)	1.19 (0.73)
*Parental rules regarding fruit consumption (%)*			
Yes	68.8	72.7	79.5
No	31.2	27.3	20.5
*Home availability of fruit %*			
Always	88.3	92.8	94.2
Not always	11.7	7.2	5.8
**Vegetables (N = 1265)**	N = 248	N = 606	N = 411
*Children’s vegetable intake, grams per day (mean, SD)*	94.0 (57.7)	100.5 (53.1)	116.9 (60.6)
*Parental vegetable intake, grams per day (mean, SD)*	148.3 (68.1)	158.4 (67.9)	176.7 (68.0)
*Parental rules regarding vegetable consumption (%)*			
Yes	83.5	85.0	92.0
No	16.5	15.0	8.0
*Home availability of vegetables*			
Always	83.1	89.4	90.5
Not always	16.4	8.0	9.5
***Breakfast (N = 1270)***	N = 246	N = 610	N = 414
*Children’s breakfast consumption (%)*			
Daily	91.9	94.3	97.3
Not daily	8.1	5.7	2.7
*Parental breakfast consumption (%)*			
Daily	83.7	91.3	95.7
Not daily	16.3	8.7	4.3

**Table 3:** **Associations between socio-economic status (SES) and children’s fruit, vegetable and breakfast consumption**

***Fruit consumption (N = 1269)***	***Multivariate regression analyses*** ^**†**^	
***SES***	***B (95% CI)***	***P-value***
Low (Ref. group)	0.84	
Intermediate	0.04 (−0.05 – 0.13)	0.38
High	**0.13 (0.04 – 0.22)**	**0.01**
***Vegetable consumption (N = 1265)***	***Multivariate regression analyses*** ^**†**^	
***SES***	***B (95% CI)***	***P-value***
Low (Ref. group)	83.89	
Intermediate	**8.33** (0.09 – 16.56)	**0.05**
High	**23.81** (14.93 – 32.69)	**0.00**
***Breakfast consumption (N = 1270)***	***Multivariate regression analyses*** ^**†**^	
***SES***	***OR (95*** **%** ***CI)***	***P-value***
Low (Ref. group)	1.00	
Intermediate	1.39 (0.78 – 2.49)	0.27
High	**2.97** (1.38 -6.39)	**0.01**

B = unstandardized coefficient, OR = Odds ratio, 95% CI = 95% Confidence Interval. Bold values represent statistically significant association. ^†^Multivariate regression analysis adjusted for: child’s age, child’s gender, child’s ethnicity and child’s BMI.

Table 4 presents data on the association between children’s SES and possible mediating variables regarding fruit consumption. Parents with a high SES consumed significantly more fruit (B = 0.25, 95% CI: 0.13-0.36), were more likely to have rules about fruit consumption (OR = 1.78, 95% CI: 1.23-2.56) and were more likely to always have fruit available at home (OR = 2.24, 95% CI: 1.25-4.00) than parents with a low SES. Parents with an intermediate SES were also more likely to always have fruit available at home than parents with a low SES (OR = 1.74, 95% CI: 1.05-2.88).

**Table 4:** **Associations between socio-economic status (SES) and the mediating variables**

***Fruit consumption (N = 1269)***	***Mediators***	***Multivariate regression analyses*** ^**†**^	
***SES***	*Parental fruit intake*	*B (95% CI)*	*P-value*
Low (Ref. group)		*0.88*	
Intermediate		0.09 (−0.02 – 0.20)	0.10
High		**0.25 (0.13 – 0.36)**	**0.00**
***SES***	*Parental rules regarding fruit intake*	*OR (95% CI)*	*P-value*
Low (Ref. group)		1.00	
Intermediate		1.18 (0.85-1.64)	0.32
High		**1.78 (1.23 – 2.56)**	**0.00**
***SES***	*Home availability of fruit*	*OR (95% CI)*	*P-value*
Low (Ref. group)		1.00	
Intermediate		**1.74 (1.05 – 2.88)**	**0.03**
High		**2.24 (1.25 – 4.00)**	**0.01**
***Vegetable consumption (N = 1265)***	***Mediators***	***Multivariate regression analyses*** ^**†**^	
***SES***	*Parental vegetable intake*	B (95% CI)	*P-value*
Low (Ref. group)		144.99	
Intermediate		**11.29** (1.24 – 21.34)	**0.03**
High		**28.86** (18.05 – 39.67)	**0.00**
***SES***	*Parental rules regarding vegetable intake*	OR (95% CI)	*P-value*
Low (Ref. group)		1.00	
Intermediate		**1.74** (1.13 – 2.69)	**0.01**
High		**2.47** (1.49 – 4.10)	**0.00**
***SES***	*Home availability of vegetables*	OR (95% CI)	*P-value*
Low (Ref. group)		1.00	
Intermediate		1.18 (0.78 – 1.77)	0.44
High		**1.93 (1.19 – 3.11)**	**0.01**
***Breakfast consumption (N = 1270)***	**Mediator**	***Multivariate regression analyses*** ^**†**^	
***SES***	*Parental breakfast intake*	*OR (95*% *CI)*	*P-value*
Low (Ref. group)		1.00	
Intermediate		**1.94 (1.24 – 3.04)**	**0.00**
High		**4.10** (2.28 – 7.37)	**0.00**

B = unstandardized coefficient, OR = Odds ratio, 95% CI = 95% Confidence Interval. Bold values represent statistically significant association. ^†^Multivariate regression analysis adjusted for: child’s age, child’s gender, child’s ethnicity and child’s BMI.

Table 5 shows that parental fruit intake, rules about fruit consumption and home availability of fruit were significantly associated with children’s fruit consumption. If parents increased their fruit consumption by one piece per day, their children increased their fruit consumption by 0.34 pieces per day. Children of parents who had fruit consumption rules were more likely to consume fruit than children of parents who had no fruit consumption rules. Children of parents who always had fruit available at home were also more likely to consume fruit than children of parents who did not always have fruit available at home.

**Table 5:** **Associations between possible mediating variables and children’s fruit, vegetable and breakfast consumption**

***Fruit consumption (N = 1269)***	***Multivariate regression analyses*** ^**†**^	
	***B (95% CI)***	***P-value***
*Parental fruit consumption*	**0.34** (0.30 – 0.39)	**0.00**
*Rules about fruit consumption*		
No (ref. group)	0.55	
Yes	**0.49** (0.42 – 0.56)	**0.00**
*Home availability of fruit*		
Not always (ref. group)	0.42	
Always	**0.48** (0.36 – 0.60)	**0.00**
***Vegetable consumption (N = 1265)***	***Multivariate regression analyses*** ^**†**^	
	***B (95% CI)***	***P-value***
*Parental vegetable consumption*	**0.46** (0.42 – 0.47)	**0.00**
*Rules about vegetable consumption*		
No (ref. group)	63.93	
Yes	**24.94** (15.20 – 34.68)	**0.00**
*Home availability of vegetables*		
Not always (ref. group)	67.66	
Always	**18.62** (9.72 – 27.51)	**0.00**
***Breakfast consumption (N = 1270)***	***Multivariate regression analyses*** ^**†**^	
	***OR (95%*** ***CI)***	***P-value***
*Parental breakfast consumption*		
Not daily (ref. group)	1.00	
Daily	**15.75** (9.04 – 27.44)	**0.00**

B = unstandardized coefficient, OR = Odds ratio, 95% CI = 95% Confidence Interval. Bold values represent statistically significant association. ^†^Multivariate regression analysis adjusted for: child’s SES, child’s age, child’s gender, child’s ethnicity and child’s BMI.

Table 6 presents the mediation analyses. In the single-mediator models, parental fruit intake explained 66.0% of the difference between children with a low SES and those with a high SES; fruit consumption rules explained 40.9% and home availability of fruit explained 23.2% of the difference in fruit intake. In the multiple-mediator models, parental fruit intake, fruit consumption rules and home availability of fruit together explained 89.5% of the difference in fruit intake between children with a low SES and those with a high SES. Parental fruit intake, fruit consumption rules and home availability of fruit had no significant mediating effect on the difference in fruit intake between children with an intermediate SES and those with a low SES.

**Table 6:** **Mediation analyses**

**Fruit consumption (N = 1269)**	***Direct association between SES and children’s fruit consumption B***	***Mediation models B (95%*** ***CI)***	***P-value***	***Percentage change***	***P-value***
***SES***		*Model A*			
Low (ref. group)	0.84	0.54			
Intermediate	0.04	0.01 (−0.07 – 0.09)	0.84	−79.49	0.36
High	0.13	0.04 (−0.09 – 0.17)	0.32	−66.01	**0.02**
***SES***		*Model B*			
Low (ref. group)	0.84	0.55			
Intermediate	0.04	0.02 (−0.06 – 0.10)	0.58	−42.16	0.42
High	0.13	0.08 (−0.01 – 0.16)	0.09	−40.85	**0.00**
***SES***		*Model C*			
Low (ref. group)	0.84	0.42			
Intermediate	0.04	0.02 (−0.07 – 0.10)	0.69	−56.10	0.40
High	0.13	**0.10 (0.01 – 0.19)**	**0.03**	−23.15	**0.02**
***SES***		*Model D*			
Low (ref. group)	0.84	0.23			
Intermediate	0.04	−0.01 (−0.08 – 0.07)	1.12	−114.44	0.40
High	0.13	0.01 (−0.07 – 0.10)	0.75	−89.53	**0.00**
***Vegetable consumption (N = 1265)***	***Direct association between SES and children’s vegetable consumption B***	***Mediation models B (95% CI)*** ^**†**^	***P-value***	***Percentage change***	***P-value***
***SES***		*Model E*			
Low (ref. group)	83.89	17.91			
Intermediate	8.33	3.19 (−3.66 – 10.05)	0.36	−61.70	0.14
High	23.81	**10.44 (2.97 – 17.93)**	**0.01**	**−56.13**	**0.00**
***SES***		*Model F*			
Low (ref. group)	83.89	63.93			
Intermediate	8.33	6.75 (−1.42 – 14.92)	0.11	−19.02	0.08
High	23.81	**21.47** (12.63 – 30.31)	**0.00**	**−9.85**	**0.00**
***SES***		*Model G*			
Low (ref. group)	83.89	67.66			
Intermediate	8.33	7.39 (−0.26 – 16.12)	0.06	−4.88	0.56
High	23.81	**22.39** (13.55 – 31.23)	**0.00**	**−5.98**	**0.00**
***SES***		Model H			
Low (ref. group)	83.89	10.57			
Intermediate	8.33	2.75 (−4.11 – 9.61)	0.43	**−**66.79	0.06
High	23.81	**9.77 (2.27 – 17.27)**	**0.01**	**−58.89**	**0.00**
***Breakfast consumption (N = 1270)***	***Direct association between SES and children’s breakfast consumption*** **OR**	***Mediation model OR (95% CI)*** ^***†***^	***P-value***	***Percentage change***	***P-value***
***SES***		*Model I*			
Low (ref. group)	1.00				
Intermediate	1.39	0.99 (0.52 – 1.90)	0.97	- 102.73	0.27
High	2.97	1.63 (0.71 – 3.67)	0.25	**- 67.89**	**0.02**

SES = socioeconomic status; B = unstandardized coefficient, OR = Odds ratio, 95% CI = 95% Confidence Interval. Bold values represent statistically significant association.

Model A: Single mediator model. This model includes the mediator ‘parental fruit consumption’.

Model B: Single mediator model. This model includes the mediator ‘parental rules regarding fruit consumption’.

Model C: Single mediator model. This model includes the mediator ‘home availability of fruit’.

Model D: Multiple mediation model. This model includes the mediators: parental fruit consumption, parental rules regarding fruit consumption and home availability of fruit.

Model E: Single mediator model. This model includes the mediator ‘parental vegetable intake’.

Model F: Single mediator model. This model includes the mediator ‘parental rules regarding vegetable consumption’.

Model G: Single mediator model. This model includes the mediator ‘home availability of vegetables’.

Model H: Multiple mediator model. This model includes the mediators: parental vegetable consumption, parental rules regarding vegetable consumption and home availability of vegetables.

Model I: Single mediator model. This model includes the mediator ‘parental breakfast consumption’.

All models are adjusted for: child’s age, child’s gender, child’s ethnicity and child’s BMI.

### Vegetable consumption

Table 2 provides data on children’s vegetable consumption and determinants of children’s vegetable consumption stratified by SES. Children with a low SES had the lowest vegetable consumption (on average 94.0 grams per day) while children with a high SES had the highest vegetable consumption (on average 116.9 grams per day).

Table 3 shows significant socio-economic differences in children’s vegetables consumption. Children with an intermediate SES and children with a high SES consumed more vegetables than children with a low SES (resp. B = 8.33, 95% CI: 0.09-16.56; B = B = 23.81, 95% CI: 14.93-32.96).

Table 4 presents data on the association between SES and possible mediating variables regarding vegetable consumption. Parents with a high SES consumed more vegetables (B = 28.86, 95% CI: 18.05-39.67), were more likely to have vegetable consumption rules (OR = 2.47, 95% CI: 1.49-4.10), and were more likely to always have vegetables available at home (OR = 1.93, 95% CI: 1.19-3.11) than parents with a low SES. Parents with an intermediate SES also consumed more vegetables (B = 11.29 95% CI: 1.24-21.34) and were more likely to have rules about vegetable consumption (OR: 1.74, 95% CI: 1.13-2.69) than parents with a low SES.

All potential mediators were significantly associated with children’s vegetable consumption (see Table 5). Children consumed more vegetables when their parents consumed more vegetables (B = 0.46, 95% CI: 0.42-0.47), when their parents had rules about vegetable consumption (OR = 24.94, 95% CI: 15.20-34.68), and when vegetables were always available at home (B = 18.62, 95% CI: 9.72-27.51).

Table 6 presents the mediation models. In the single-mediator models, parental vegetable intake explained 56.1% of the difference in vegetable consumption between children with a low SES and those with a high SES; vegetable consumption rules explained 9.9% and home availability of fruit explained 6.0%. In the multiple-mediator model, all the mediators together explained 58.89% of the difference in vegetable intake between children with a low SES and those with a high SES. Parental vegetable intake, vegetable consumption rules and home availability of vegetables had no significant mediating effect on the difference in vegetable intake between children with an intermediate SES and those with a low SES.

### Breakfast consumption

Table 2 presents data on children’s and parents breakfast consumption. Children and parents with a high SES more often reported to have breakfast on a daily basis than children and parents with a low and intermediate SES. Table 3 reports on the association between SES and children’s breakfast consumption. Children with a high SES were more likely to eat breakfast on a daily basis than children with a low SES (OR = 2.97, 95% CI: 1.38-6.39). There was no significant difference in breakfast consumption between children with an intermediate SES and those with a low SES.

Parents with high and intermediate SES were more likely to consume breakfast on a daily basis than parents with a low SES (see Table 4). Table 5 shows that children were more likely to eat breakfast on a daily basis when their parents ate breakfast on a daily basis (OR = 15.75, 95% CI: 9.04-27.44). Table 6 shows the final mediation model; parental breakfast consumption explained 67.9% of the differences in breakfast consumption between children with a high SES and those with a low SES. Parental breakfast consumption had no significant mediating effect on the difference in breakfast consumption between children with an intermediate and with a high SES.

## Discussion

The first aim of this study was to examine the association between SES and children’s fruit, vegetable and breakfast consumption. We found that children with a high SES consumed more fruit and vegetables and consumed more often breakfast on a daily basis, than children with a low SES. These findings are in line with those from the majority of similar studies [9,13-15,17,19,35] and emphasise that children from low socio- economic groups can be considered an important target for interventions to improve dietary behaviour.

However, maternal education level, (and other measures of SES), are not considered to have a direct effect on dietary behaviour and are not easily modifiable. To explain socio-economic disparities in children’s dietary behaviour, several studies examined socio-economic differences in the home food environment of children. These studies showed that the home food environment of children of mothers with a low educational level was less supportive than the home food environment of children of mothers with a high educational level [36,37]. For example, adolescents of mothers with a low educational level were more likely to report that unhealthy foods were always or usually available at home, while adolescents of mothers with a high educational level were more likely to report that fruit was always or usually available at home and that vegetables were always served at dinner time [38]. In addition, a study among 5–6 year old children found comparable results; households of mothers with a low educational level were more likely to watch television while eating dinner and mothers with a low educational level were more likely to have negative perceptions about the quality and variety of fresh fruit and vegetables at their local shops [39]. Furthermore, Hupkens *et al.* found that mothers with a high educational level more often limited their children’s intake of unhealthy foods (e.g. sweets, soft drinks, chips). These differences in the number of restricted foods by educational level were partly explained in health and taste considerations between mothers with a low and high educational level [40]. A more recent study also found socio-economic differences in food parenting practices; frequent consumption of fruit and vegetables, restrictive rules, verbal praise, negotiation and restrain from negative modelling were all more common among mothers with a high educational level [41]. The present study also shows that aspects of the home food environment differed by SES, where low SES had the less supportive home environment.

However, socio-economic differences in determinants of the home food environment do not necessarily account for socio-economic differences in children’s dietary behaviour. Therefore, a second aim of this study was to investigate modifiable factors of the home food environment that mediate the association between SES and children’s fruit, vegetable and breakfast consumption. We included parental intake, home availability and parental rules about children’s fruit and vegetable consumption as possible mediating variables in the association between SES and children’s fruit and vegetable intake. Our results indicate that all the studied home environmental factors mediate the association between SES and children’s fruit and vegetable intake. Moreover, our results indicate that the difference in fruit and vegetable consumption between children with a low and high SES is explained in particular by parental intake of fruit and vegetables. Very few studies have assessed mediators of the association between socio-economic status and children’s fruit and vegetables intake. Vereecken *et al.* found that differences in children’s fruit and vegetable consumption by mother’s educational level were completely explained by mother’s consumption and parenting practices [41]. Bere *et al.* concluded that home accessibility was the strongest mediator of the association between maternal educational level and adolescent’s fruit and vegetable consumption [13]. Furthermore, Hilsen *et al.* also found that accessibility of fruit and vegetables mediates part of the association between socio-economic status and adolescent’s fruit and vegetable intake [17]. In addition, they found that accessibility of fruit and vegetables explains part of the increase in SES disparities in fruit and vegetable consumption between 2001 and 2008.

To our knowledge, ours is the first study to assess possible explanatory variables of socio-economic disparities in children’s breakfast consumption. We found that the difference in breakfast consumption between high SES children and low SES children was mediated by parental breakfast consumption. However, we included only one possible mediator in our analyses (parental breakfast consumption), while other potentially mediating variables were not included. For example, parenting practices are associated with children’s breakfast consumption and may also be an explanatory variable of socio-economic differences in children’s breakfast consumption.

It is known that aspects of the home environment are associated with children’s dietary behaviour. This study indicates that home environmental factors also play a role in the explanation of socio-economic disparities in children’s healthy eating behaviour. Given that parental intake was the strongest mediator and that parents shape the home food environment (e.g. they decide which food is available at home and can set food rules), parents play an important role in the development of children’s dietary behaviour. Therefore, parents can be important targets for interventions. Moreover, it is necessary to reach parents with a low SES and to increase their own consumption of fruit, vegetables and breakfast, to increase the home availability of healthy products and to set food rules for their children. Campbell *et al.* found that maternal nutrition knowledge was associated with children’s fruit and vegetable consumption and also with the home availability of fruit and vegetables [42]. Therefore, targeting parental nutritional knowledge (especially among those with a low educational level) may be an effective way to improve the home food environment. Besides interventions that aim at the importance of family involvement, also multiple-setting interventions are effective in changing children’s dietary behaviour. In the latter case, children receive the messages in more than one setting (e.g. at home, school, and the sports club) thereby increasing the chance that such an intervention will be more effective than a single-setting approach [43]. However, interventions aiming to improve children’s dietary behaviour, such as children’s fruit and vegetable consumption are also necessary for children from higher socio-economic backgrounds since the majority of all children (including children of higher educational background) does not consume the recommended amount of fruit and vegetables.

The present study has some limitations. First, this study has a cross-sectional design, which does not allow to draw conclusions about causal relationships. However, as educational level is a consistent factor over time, it is highly unlikely that children’s food consumption will affect a mother’s educational level. Although it is possible that children’s fruit and vegetable consumption contributes to the amount of fruit and vegetables available at home, or to parental consumption rules regarding fruit and vegetables, we believe that the impact of the home availability and the consumption rules of fruit and vegetables on children’s fruit and vegetable intake are larger. Therefore, we expect the directions of the associations we found to be as presented in Figure 1. Second, assessments of child’s fruit, vegetable and breakfast consumption were based on parent’s reports instead of child reports. Child reports might be more valid, although this remains unclear. Nevertheless, Tak *et al.* [44] concluded that parents’ reports could be considered as a valid method to measure children’s fruit and vegetable consumption, although the use of parent’s reports may evoke socially desirable answers. Finally, we measured breakfast frequency and not breakfast quality, which is associated with the nutrient adequacy of diets [45].

## Conclusion

This study shows that children of mothers with low educational level have less healthy eating habits than children of mothers with a high educational level. Our study adds to the knowledge on possible mechanisms underlying socio-economic differences in healthy eating behaviour of children. Parent’s food intake, home availability of healthy foods and parental rules about children’s fruit and vegetable intake mediated the association between maternal educational level and children’s healthy eating behaviour. Interventions to improve children’s dietary behaviour and to reduce socio-economic disparities in children’s eating habits, may benefit by focusing on the role of parents in the development of children’s dietary behaviour.
